# Numerous scattered clusters of submillimeter blue-gray and brown macules on the skin of a patient with progressive joint destruction

**DOI:** 10.1016/j.jdcr.2026.09.010

**Published:** 2026-09-12

**Authors:** Graham Grisedale, Andrew Siref, Ryan Trowbridge

**Affiliations:** Division of Dermatology, Creighton University School of Medicine, Omaha, Nebraska

**Keywords:** alkaptonuria, dermoscopy, ochronosis, pigmented basal cell carcinoma

## Clinical images

### Case description

A man in his early 40s with a history of dysplastic nevi was referred to dermatology for a total body skin examination and was found to have numerous scattered clusters of submillimeter blue-gray and brown macules on his upper extremities (Fig 1). Further skin examination revealed blue-gray ill-defined patches involving the pinna. In discussion, the patient reported previously being highly physically active, but described progressive, unexplained joint deterioration, especially of the hips, over the past decade.

**Fig 1:**
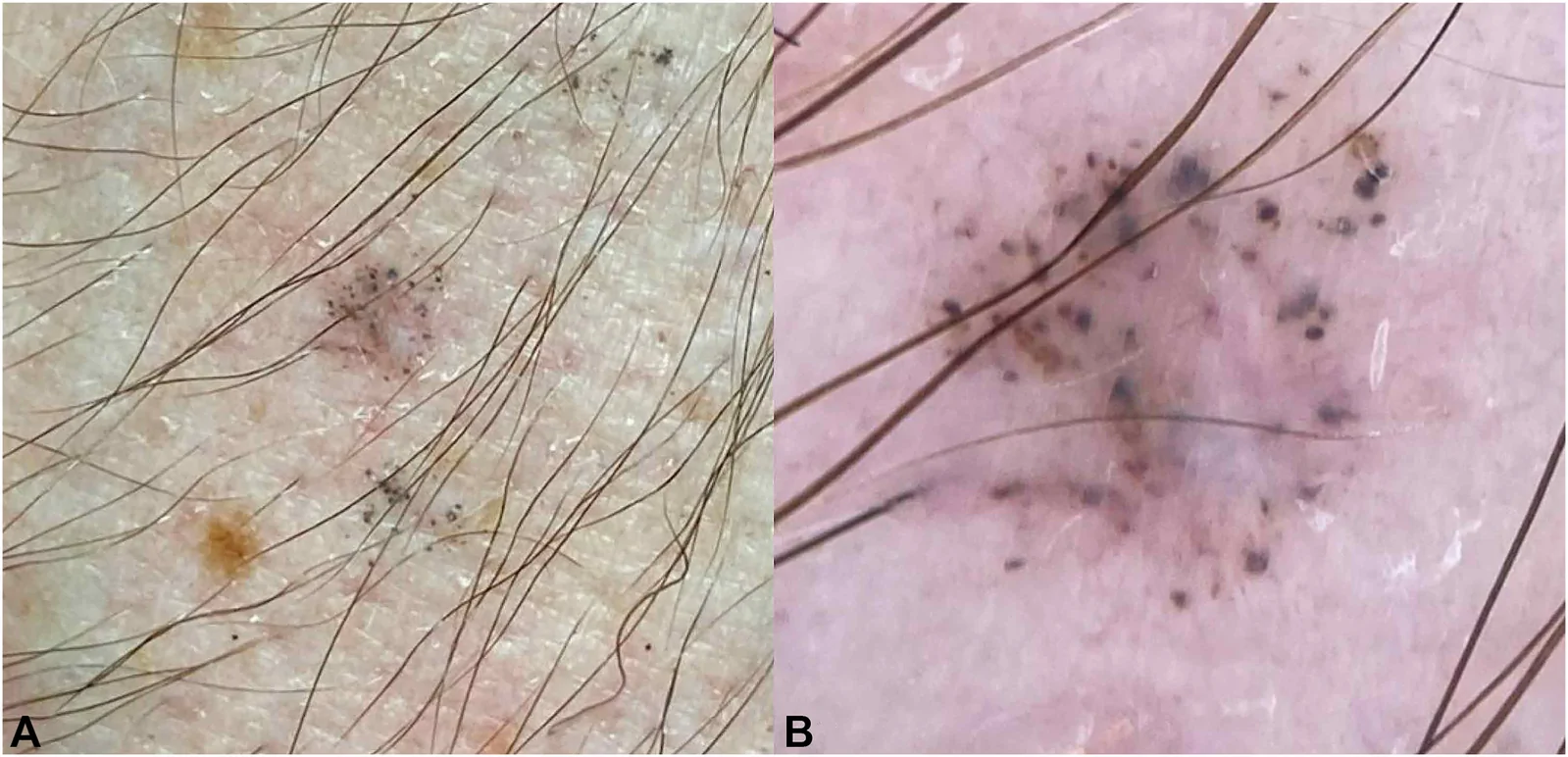
(**A**) Gross photographic image. (**B**) Polarized dermoscopy.

**Question: Which is the most likely diagnosis for his skin lesions?**

**A.** Basal cell carcinoma.

**B.** Ochronosis.

**C.** Melanoma.

**D.** Pigmented actinic keratosis.

**E.** Dysplastic nevi.

*Answer*: B. Ochronosis.

## Discussion

Alkaptonuria is a genetic enzyme deficiency of homogentisate 1,2-dioxygenase affecting approximately 1 in 100,000 to 250,000 individuals.^1^ Deficiency of this enzyme leads to accumulation of homogentisic acid, which oxidizes into a dark blue-gray pigment that deposits in connective tissues, called ochronosis. Skin findings often provide the earliest clinical clue, prompting confirmation through urine testing (Fig 2), chromatography, or genetic analysis of the *HDG* gene on chromosome 3.^2^

**Fig 2:**
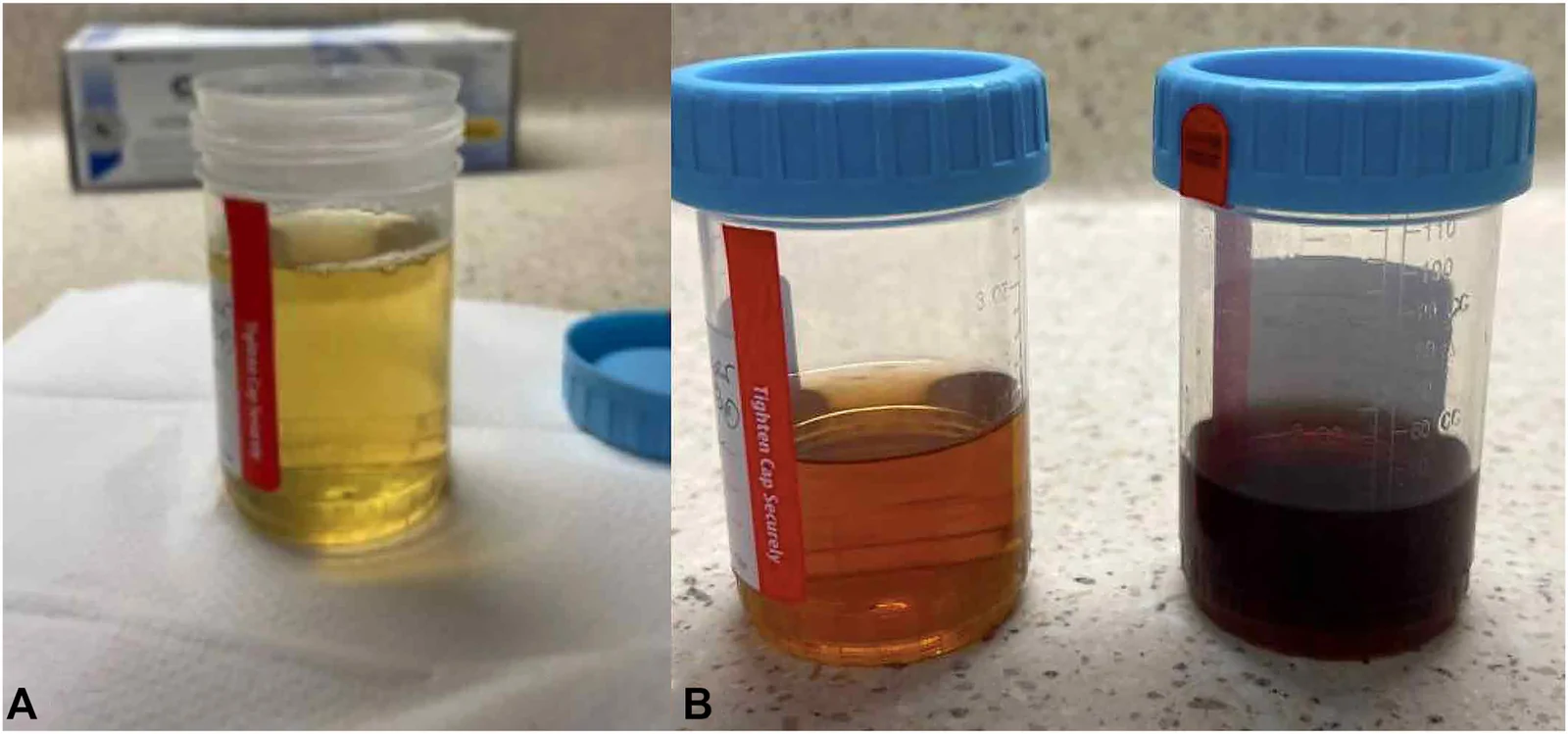
A simple bedside confirmatory test includes observation of oxidation of a urine sample demonstrating a distinct color change. Patient’s urine (**A**) at collection and (**B**) 16 hours after collection without (left) and with (right) potassium hydroxide.

Pigment deposition traditionally occurs in the skin, sclera, cartilage, and joints, leading to premature degenerative arthropathy seen in this patient.^3^ The cutaneous findings in this patient, clusters of blue-gray and brown submillimeter macules, is a unique finding that has not been reported in endogenous ochronosis. There are some similarities between our dermoscopic observations and those previously described in exogenous ochronosis, primarily the presence of blue-gray and brown globules.^4^ However, the confluent bilateral speckled gray facial pigmentation described by Gil et al^4^ differs from the clustered findings in this case in both anatomic distribution (face vs extremities) and spatial organization (broad confluent patches vs small discrete clusters). Dermoscopically, Gil et al^4^ demonstrated prominent annular and arciform perifollicular structures, which were not prominent in this case. A 4-mm punch biopsy confirmed the diagnosis of ochronosis (Fig 3).

**Fig 3:**
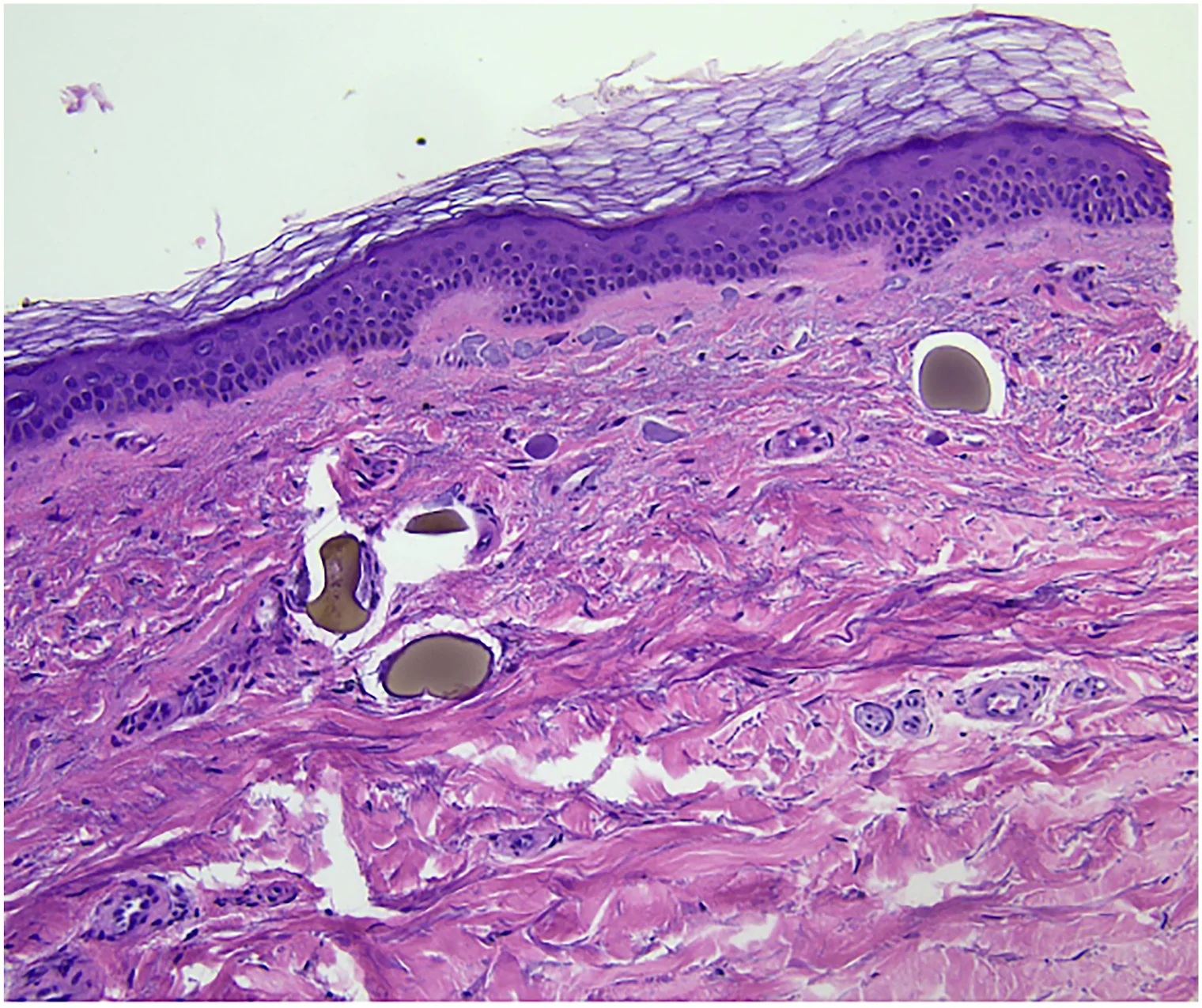
Pathologic examination revealed normal epidermal and dermal architecture with brown-yellow pigment in the superficial dermis; Verhoeff elastic stain did not highlight the pigment, and elastic fibers were unremarkable; no evidence of malignancy was observed.

Recognizing how exogenous ochronosis resembles and differs from basal cell carcinoma is essential. While these globules closely resemble those seen in basal cell carcinoma when viewed in isolation, the blue-gray ovoid nests, dots, and globules typically seen in basal cell carcinoma are accompanied by characteristic vascular patterns, punctate erosions, and myxoid stromal changes (Fig 4), and individual lesions are typically far less numerous.

**Fig 4:**
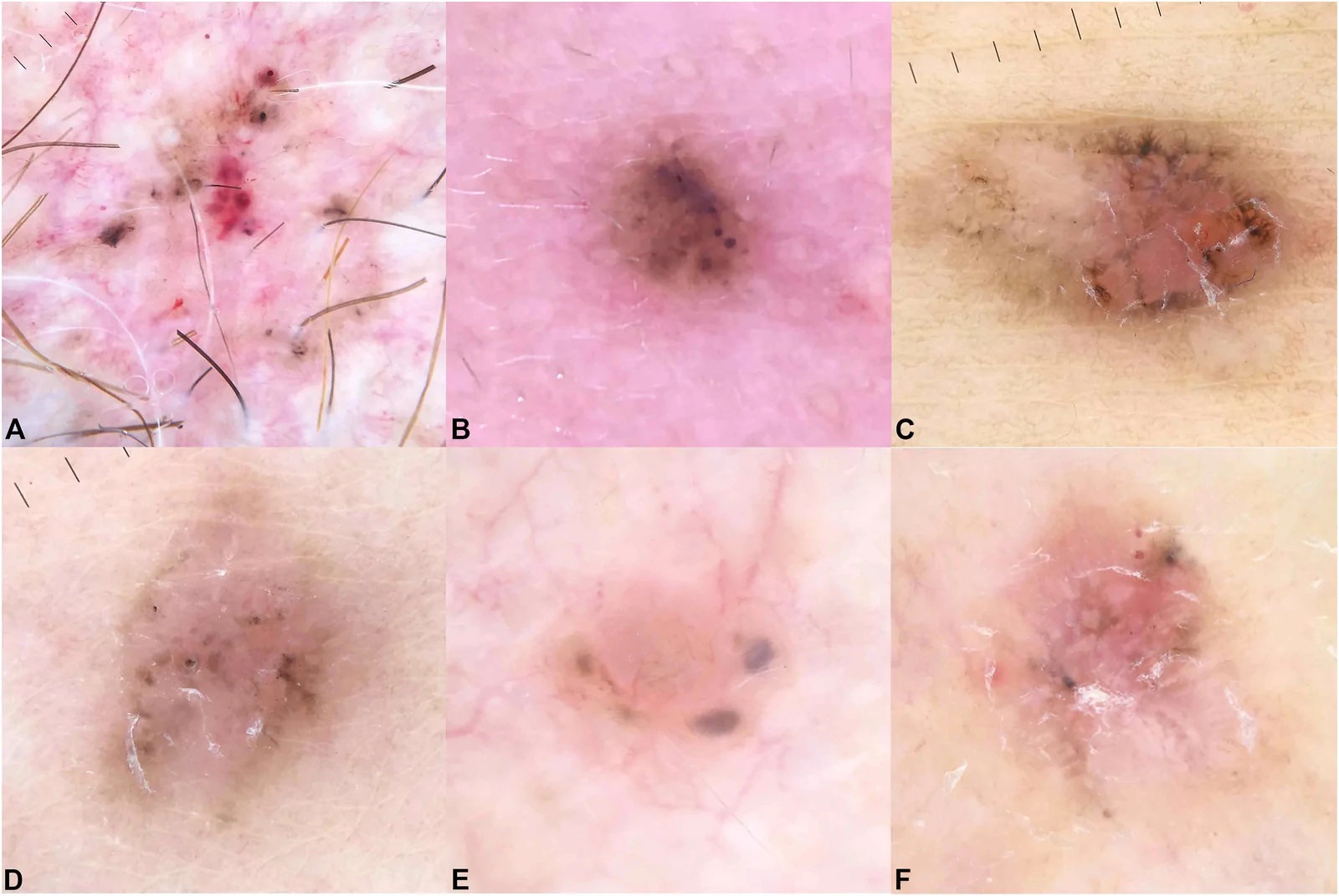
(**A**-**F**) Examples of characteristic pigmented dermoscopic findings of basal cell carcinoma for comparison.

This case adds a unique clinical and dermoscopic observation, which may represent an earlier and subtler cutaneous manifestation of alkaptonuria. Careful clinical examination of this subtle finding may facilitate earlier recognition of a disease that can ultimately lead to severe joint destruction and impaired quality of life.
